# Role of viral hemagglutinin glycosylation in anti-influenza activities of recombinant surfactant protein D

**DOI:** 10.1186/1465-9921-9-65

**Published:** 2008-09-23

**Authors:** Kevan L Hartshorn, Richard Webby, Mitchell R White, Tesfaldet Tecle, Clark Pan, Susan Boucher, Rodney J Moreland, Erika C Crouch, Ronald K Scheule

**Affiliations:** 1 Boston University School of Medicine, Department of Medicine, Boston MA, USA; 2 Department of Infectious Diseases, St Jude Children's Research Hospital, Memphis, TN, USA; 3 Genzyme Corporation, Framingham, MA, USA; 4 Department of Pathology and Immunology, Washington University School of Medicine, St. Louis, MO, USA

## Abstract

**Background:**

Surfactant protein D (SP-D) plays an important role in innate defense against influenza A viruses (IAVs) and other pathogens.

**Methods:**

We tested antiviral activities of recombinant human SP-D against a panel of IAV strains that vary in glycosylation sites on their hemagglutinin (HA). For these experiments a recombinant version of human SP-D of the Met11, Ala160 genotype was used after it was characterized biochemically and structurally.

**Results:**

Oligosaccharides at amino acid 165 on the HA in the H3N2 subtype and 104 in the H1N1 subtype are absent in collectin-resistant strains developed *in vitro *and are important for mediating antiviral activity of SP-D; however, other glycans on the HA of these viral subtypes also are involved in inhibition by SP-D. H3N2 strains obtained shortly after introduction into the human population were largely resistant to SP-D, despite having the glycan at 165. H3N2 strains have become steadily more sensitive to SP-D over time in the human population, in association with addition of other glycans to the head region of the HA. In contrast, H1N1 strains were most sensitive in the 1970s–1980s and more recent strains have become less sensitive, despite retaining the glycan at 104. Two H5N1 strains were also resistant to inhibition by SP-D. By comparing sites of glycan attachment on sensitive vs. resistant strains, specific glycan sites on the head domain of the HA are implicated as important for inhibition by SP-D. Molecular modeling of the glycan attachment sites on HA and the carbohydrate recognition domain of SPD are consistent with these observations.

**Conclusion:**

Inhibition by SP-D correlates with presence of several glycan attachment sites on the HA. Pandemic and avian strains appear to lack susceptibility to SP-D and this could be a contributory factor to their virulence.

## Introduction

Influenza A viruses (IAVs) remain a major cause of morbidity and mortality and have the potential to cause massive mortality if novel pandemic strains emerge through reassortment of human and avian strains or direct adaptation of avian strains to humans (as appears to have occurred in 1918) [1,2]. Certain groups of subjects are known to be more susceptible to adverse effects of influenza infection, including the very young and old, and subjects with a variety of co-morbid conditions [3]. It is likely that innate host defense mechanisms play an important role in defense against novel strains associated with antigenic drift or shift. These defense mechanisms may play a greater role in more vulnerable subjects (e.g., those who are immunocompromised in other ways).

We and others have extensively studied the role of collectins in the early defense against influenza [4-8]. Mice lacking either SPA or SP-D experience greater viral replication, inflammation and illness in the first several days after infection with IAV strains known to be susceptible to inhibition by these host defense lectins *in vitro *[4,9,10]. Host defense against IAV can be restored in these mice by instillation or over-expression of wild type forms of rat SP-D. Removal of SP-D from human bronchoalveolar lavage fluid strongly reduces its ability inhibit recent human strains of IAV [5].

There are several polymorphic forms of SP-D. One of these involves a variation between Thr and Met at amino acid 11 in the N-terminus and another between Ala and Thr at amino acid 160. The identity of the amino acid 11 affects serum levels and resistance to infections (e.g., respiratory syncytial virus or Mycobacterium tuberculosis) *in vivo *[11-13]. Individuals homozygous for the Thr11 form of SP-D (~18% in several studies) have lower serum levels of SP-D and a greater proportion of their SP-D is in the form of trimers as compared to individuals with the Met11 form [14]. Natural trimers isolated from amniotic fluid have been shown to have reduced binding affinity for IAV [14] and a reduced ability to neutralize the virus or to potentiate neutrophil respiratory burst responses to IAV [15]. The functional significance of the Thr/Ala160 polymorphism is unclear. Most of our prior studies using recombinant SP-D have involved the Thr11, Thr160 form. In this study we test the antiviral activities of the Met11, Ala160 form.

SP-D (and other collectins including mannose binding lectin (MBL), conglutinin and CL43) inhibits IAV by binding to glycans on the viral envelope proteins. Binding of these collectins to high mannose oligosaccharides on the hemagglutinin (HA) appears to be most important for viral neutralization [16-18]. Of interest, more recent strains of IAV of the H3N2 and H1N1 human lineages show greater susceptibility to inhibition by SP-D or MBL in conjunction with increasing glycosylation of the HA [19]. The more recent strains also show reduced virulence in mice. Similar findings were obtained in an elegant study involving reverse genetics to add or remove glycans from the HA of the human H3N2 subtype [20].

The goals of the current study were to evaluate antiviral activities of recombinant full length SP-D of the Met11, Ala160 form and to determine the impact of specific glycosylation sites on the HA of various subtypes of influenza on susceptibility to inhibition by SP-D.

## Materials and methods

### Virus Preparations

Influenza strains were grown in the chorioallantoic fluid of ten day old chicken eggs and purified on a discontinuous sucrose gradient as previously described [21]. The virus was dialyzed against PBS to remove sucrose, aliquoted and stored at -80°C until needed. Philippines 82/H3N2 (Phil82), Phil82/BS, Brazil 78 H1N1 (Braz78), Braz78/BS, and Mem71H-BelN/H3N1 strains were kindly provided by Dr. E. Margot Anders (Univ. of Melbourne, Melbourne, Australia). Post thawing the viral stocks contained ~5 × 10^8 ^plaque forming units/ml. Other strains were prepared and tested at St. Jude's Hospital.

### SP-D preparations

Recombinant human SP-D having a Met at amino acid 11 (and Ala at amino acid 160) was prepared as described [22,23]. CHO DHFR cells were used for these preparations. Superdex was used for the affinity column instead of maltose agarose for these preparations. Stock concentrations of rhSP-D were 0.5 mg/ml in buffer (20 mM Tris, 200 mM NaCl, l mM EDTA pH 7.4). The endotoxin level of all SP-D preparations was 0.1~0.5 EU/ml (Limulus Lysate Assay, Cambrex, Walkersville, MD).

### SP-D characterization

The SP-D preparations were characterized by matrix-assisted laser desorption/ionization-time-of-flight mass spectroscopy (MALDI-TOF MS), SDS PAGE, analytical ultracentrifugation (AUC), size-exclusion chromatography-multi angle light scattering (SEC-MALS), and atomic force microscopy (AFM). Only the AUC and AFM characterization results are shown here.

Sedimentation velocity characterization of rhSP-D was performed on a Beckman Optima XLA analytical ultracentrifuge at 40 K rpm, 20°C using UV detection. Samples were at 50 μg/ml in 10 mM HEPES, 150 mM NaCl, 2 mM EDTA, pH 7.5. Data analysis was performed using SedFit v9.4 with a confidence level set at 0.95. Results are shown as a continuous distribution to represent the various molecular species in each sample.

Atomic force microscopy characterization of rhSP-D was carried out on a NanoScope instrument (Veeco Instruments, Inc., Plainview, NY) using a scan size of 1 μm and a scan rate of 1.001 Hz. Samples were placed on fresh mica grids and images obtained under aqueous phase conditions using tapping mode.

### Hemagglutination (HA) inhibition assay

HA inhibition was measured by serially diluting SP-D preparations in round bottom 96 well plates (Serocluster U-Vinyl plates; Costar, Cambridge, MA) using PBS containing calcium and magnesium as a diluent [5]. After adding 25 μl of IAV, giving a final concentration of 40 HA units per ml or 4 HA units/well, the IAV/protein mixture was incubated for 15 min. at room temperature, followed by addition of 50 μl of human type O (in Table 1) or an avian (in Table 2; note turkey and chicken gave identical results) erythrocyte suspension. The minimum concentration of protein required to fully inhibit the hemagglutinating activity of the viral suspension was determined by noting the highest dilution of protein that still inhibited hemagglutination. Inhibition of HA activity in a given well is demonstrated by absence of formation of an erythrocyte pellet. If no inhibition of HA activity was observed at the highest protein concentration used then the value is expressed as > the maximal protein concentration.

**Table 1:** HA inhibition of wild type and bovine serum resistant strains of IAV by Met11 rhSP-D

**Viral Strain**	**Description**	**HA inhibiting concentration(ng/ml)**
Phil82	H3N2 wild type HA	9 ± 3
Phil82/BS	Lacks glycan 165 on HA	63 ± 3*
Braz78	H1N1 wild type HA	47 ± 13
Braz78/BS	Lacks glycan at 104 on HA	253 ± 22*
Mem71H-BelN	Recombinant H3N1 strain	43 ± 9**

Results are mean ± SEM of three experiments with human type O negative erythrocytes.

• = p < 0.04 compared with parental viral strain

• ** p < 0.05 compared with Phil82 strain

**Table 2:** Sensitivity of different influenza strains to inhibition by SP-D and their HA glycan attachments

**H3N2**	**SPD**	63	81	**122**	126	**133**	**144**	165	246	276
X31 (Aichi/68xA/PR8)	>2 μg		+					+		
A/Udorn/72	>2 μg		+					+		
A/Shandong/9/93	>2 μg	+			+			+	+	+
A/Wuhan/359/95	>2 μg	+			+			+	+	
A/Sydney/5/97	62 ng	+		+	+	+		+	+	
A/Panama/2007/99	7.8 ng	+		+	+	+	+	+	+	
A/Fujian/411/02 (rg)	3.9 ng	+		+	+	+	+	+	+	
***Sialic acid distance *(Å)**		*23*	*28*	*20*	*18*	*7.6*	*9.8*	*20*	*19*	*43*

										
			
**H1N1**	**SPD**	71	104	142	**144**	**172**	177			
			
A/Puerto Rico/8/34	>2 μg									
A/Memphis/10/78	3.9 ng		+		+	+	+			
A/Memphis/7/80	7.8 ng		+		+	+	+			
A/Memphis/14/96	1 μg	+	+	+			+			
A/New Caledonia/20/99	>2 μg	+	+	+			+			
A/Memphis/6/2001	1 μg	+	+	+			+			
***Sialic acid distance *(Å)**		*21*	*17*	*15*	*9*	*13*	*24*			
			
										
							
**H1N1**	**SPD**	170	181							
							
A/HK/213/03	2 μg		+							
A/Vietnam/1203/04	>2 μg	+	+							
***Sialic acid distance *(Å)**		*15*	*27*							

Notes:

1. SPD masses refer to amount required in hemagglutination inhibition assay to inhibit influenza-mediated agglutination of chicken and turkey red blood cells.

2. Numbers refer to amino acid number in the influenza HA "head"; "+" indicates potential N-linked glycosylation site based on sequence. **Bold **numbers indicate residues with large apparent influence on interaction of virus with SP-D

### Measurement of viral aggregation

Viral aggregation was measured by assessing light transmission through stirred suspensions of IAV as previously described [24].

### Fluorescent focus assay of IAV infectivity

MDCK cell monolayers were prepared in 96 well plates and grown to confluency. These layers were then infected with diluted IAV preparations for 45 min. at 37°C in PBS and tested for presence of IAV infected cells after 7 hrs. using a monoclonal antibody directed against the influenza A viral nucleoprotein (provided by Dr. Nancy Cox, CDC, Atlanta, GA) as previously described [25]. IAV was pre-incubated for 30 min. at 37°C with SP-D or control buffer, followed by addition of these viral samples to the MDCK cells.

### Neuraminidase (NA) inhibition assay

NA activity of IAV was measured by an enzyme-linked micro plate assay in which *Arachis hypogaea *peanut lectin was used to detect β-D-galactose-N-acetylglucosamine sequences exposed after the removal of sialic acid from fetuin [26]. Wells of microtiter plate were coated with 50 μl of fetuin (Sigma F-2379: 20 μl/ml in PBS) overnight at 4°C and washed with PBS. Dilutions of IAV strains with different concentrations of SP-D were preincubated for 30 min. at 37°C and 50 μl of the mixture was added to fetuin coated wells and incubated at 37°C for 2 hrs. After the wells were washed, 50 μl of peroxidase labeled peanut lectin [Sigma L-6135: 20 μl/ml in BSA (0.5%)] was added to each well for 30 min. at room temperature, followed by washing and incubation with 50 μl of TMP-peroxidase (Bio-Rad Labs, Hercules, CA) for 20 min. Finally, 50 μl of 1N H_2_SO_4 _was added to the wells and the absorbance was measured by ELISA plate reader at 450 nm.

### Human Neutrophil Preparation

Neutrophils from healthy volunteers were isolated to > 95% purity by using dextran precipitation, followed by Ficoll-Paque gradient separation for the removal of mononuclear cells, and then hypotonic lysis to eliminate any contaminating erythrocytes, as previously described [21]. Cell viability was determined to be >98% by trypan blue staining. The isolated neutrophils were resuspended at the appropriate concentrations in control buffer (PBS) and used within 2 hours. Neutrophil collection was done with informed consent as approved by the Institutional Review Board of Boston University School of Medicine.

### Measurement of IAV uptake by neutrophils

Fluorescein isothiocyanate (FITC)-labeled IAV (Phil82 strain) was prepared and uptake of virus by neutrophils was measured by flow cytometry as described [24,27]. In brief, IAV was incubated with neutrophils for 30 minutes at 37°C in presence of control buffer. Trypan blue (0.2 mg/ml) was added to these samples to quench extracellular fluorescence. Following washing, the neutrophils were fixed with paraformaldehyde and neutrophil associated fluorescence was measured using flow cytometry. The mean neutrophil fluorescence (>1000 cells counted per sample) was measured.

### Statistics

Statistical comparisons were made using Student's paired, two-tailed *t *test or ANOVA with post hoc test (Tukey's). ANOVA was used for multiple comparisons to a single control.

## Results

### Size characterization of Met11 recombinant human SP-D

**Figure 1:**
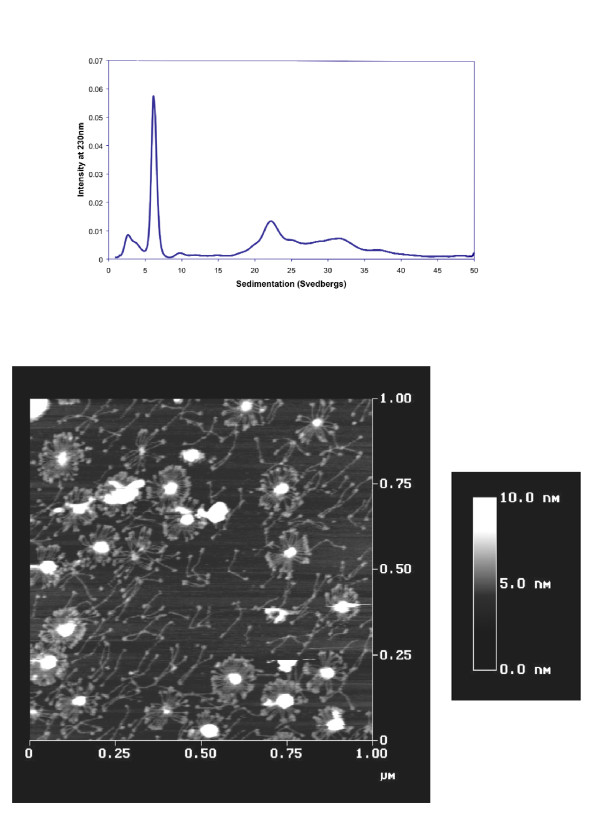
**Size distributions of Met11, Ala160 SP-D**. Molecular size distribution of an unfractionated Met11 rhSP-D preparation was determined using (A) analytical ultracentrifugation, and (B) atomic force microscopy. Both methods show a distribution of trimers, dodecamers and higher molecular weight multimers.

Figure 1A shows the size distribution of an unfractionated preparation of Met11 rhSP-D characterized by AUC. The SP-D contains small amounts of trimers (~3 Sv) together with larger amounts of dodecamers (~6.5 Sv) and high molecular weight multimers (20–35 Sv). These AUC results were confirmed by atomic force microscopy (AFM), ie., small amounts of trimers can be seen interspersed with dodecamers (cruciform structures) and higher order multimers (Figure 1B). The headgroups of SP-D, which contain the carbohydrate recognition domain, can be seen easily, especially on the periphery of the radially symmetric multimers.

### Antiviral activities of the Met11 form of recombinant human SP-D

**Figure 2:**
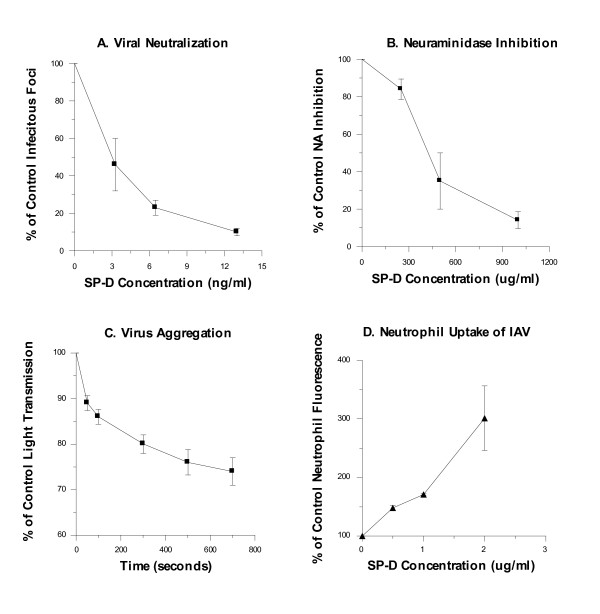
**Antiviral activities of Met11 SP-D**. (A) SP-D inhibited infectivity of the Phil82 IAV strain. SP-D also (B) inhibited neuraminidase activity, (C) caused viral aggregation, and (D) increased viral uptake by neutrophils. All results are mean ± SEM of at least three independent experiments.

Figure 2 demonstrates that the antiviral activities of this Met11 SP-D are comparable to those we have previously reported for dodecamers of Thr11 SP-D [28]. The IAV strain used in these assays was the wild type human Phil82 strain of the H3N2 subtype. In brief, the Met11 SP-D inhibited infectivity and NA activity and caused viral aggregation. Furthermore, Met11 SP-D increased neutrophil uptake of IAV.

### Activity of SP-D against bovine serum inhibitor resistant strains of IAV

As shown in Table 1, Met11 SP-D also strongly inhibited HA activity of the Phil82 strain for human Type O erythrocytes. By comparison, SP-D caused less inhibition of the Phil82/BS strain. The Phil82/BS strain was derived by culturing the parental strain in presence of bovine serum inhibitors (later shown to largely be the collectin, conglutinin) [29]. The Phil82/BS strain lacks an oligomannose glycan on the tip of the HA at position 165. Of note, however, loss of this glycan did not eliminate sensitivity to Met11 SP-D, implying that other glycans on the HA head may mediate binding to SP-D as well. A similar strain was derived from Brazil 78 H1N1 (Braz78) by growth in presence of bovine serum (called Braz78/BS). Compared to the parental strain, the Braz78/BS strain lacks an oligosaccharide attachment at residue 104 on the HA [29]. The Braz78/BS strain required significantly higher concentrations of Met11 SP-D for inhibition than the Braz78 parental strain; however, as in the case of Phil82/BS, Braz78/BS was inhibited indicating that glycans other that at 104 can mediate binding and inhibition by SP-D. The Mem71_H_-Bel_N_/H3N1 strain was significantly less sensitive to inhibition by SP-D than Phil82. these results led us to try to determine which glycan attachments on the HA of H1 or H3 viral strains are important in binding SP-D, apart from the important ones at 165 on H3 and 104 on H1.

#### Chronological analysis of HA glycosylation and susceptibility to inhibition by SP-D

**Figure 3:**
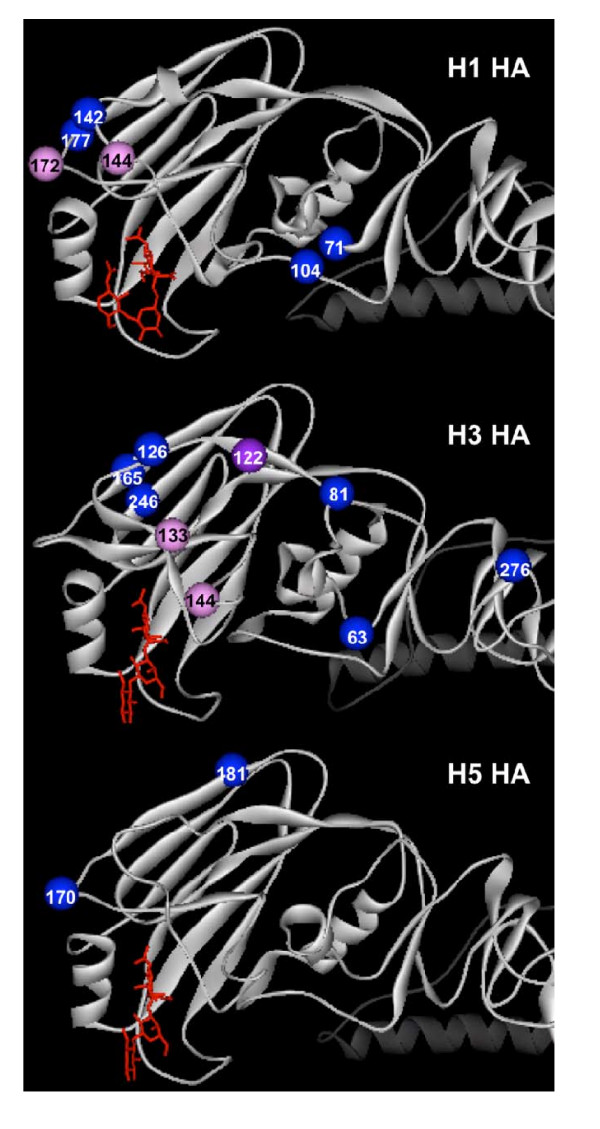
**Location of added N-linked glycans on the HA head domains of H3, H1 and H5 hemagglutinins**. The structures of the H1, H3, and H5 HA monomer head regions are shown as white ribbons with the Ca atoms of the corresponding glycosylation sites listed in Table 2 highlighted as colored balls. PDB1RVZ coordinates of the 1934 H1N1 HA complexed with LSTc trisaccharide [31], PDB1HGG coordinates of the X31 H3N2 HA complexed with (a2-3)sialyllactose [38], and PDB12FK0 coordinates of the 2004 Vietnamese H5N1 HA [39] were displayed using Accelrys DS Visualizer 1.7. The trisaccharide (a2-3)sialyllactose was modeled into the H5N1 structure by superpositioning the H5N1 structure onto that of H3N2 structure complexed with (a2-3) sialyllactose to illustrate the primary sugar binding site on HA. Increased sensitivity to inhibition by SP-D appears to correlate with acquisition of certain added glycans on the HA head region (see Table 2). The Ca atoms of these sensitive glycan positions are depicted as magenta balls while the other glycan Ca atoms are shown as blue balls. Although the glycan at 122 in H3 (purple ball) was identified as potentially important based on hemagglutination inhibition, it is significantly further removed from the sialic acid binding site (and therefore less likely to be important) than the glycans at 133 and 144. The trisaccharide sugars are depicted as red sticks.

Table 2 shows concentrations of SP-D needed to inhibit chronologically listed H3N2, H1N1, and H5N1 IAV strains. The table also shows the sites of N-linked glycosylation on the HA "head" of these strains. Note that all H3N2 strains have a glycan at 165 [30]. The parental Phil82 strain has glycans at 63, 126, 144, 165, and 246 on the HA head. There is a progressive increase in glycan attachments and susceptibility of H3N2 strains to SP-D over time. The Aichi68 strain (with HA of the initial pandemic strain) has attachments only at 81 and 165 and was resistant to inhibition by SP-D in these assays (at least up to 2 μg/ml). Based on a correlation of SP-D sensitivity and glycan position, glycan attachments at 122, 133, and 144 appear to be most strongly associated with sensitivity to SP-D. Because glycans 122 and 133 appear together chronologically and correlate with increased sensitivity, at least one of these positions would appear to be important for SP-D binding. Figure 3A shows the location of these three sites with respect to the sialic acid binding site of the HA. Amino acids 133 and 144 in particular are 7.6 and 9.8 Angstroms distant from the sialic acid binding site, and may therefore play a more important role in neutralization by SP-D than residues 122 and 165, which are 20A distant.

A somewhat different chronological pattern is evident with the H1N1 isolates (Table 2). H1 numbering was used for denoting glycan attachment sites in Table 2. In general there is a substantial increase in glycosylation on the H1 HA head region comparing strains from early in the 20^th ^century (e.g., see PR-8 strain) and those circulating since the re-introduction of H1N1 in 1977. The PR-8 strain and the 1918 pandemic strain [31] had no glycan sites on the HA head, and PR-8 is fully resistant to SP-D. Although we could not test them, the 1918 strains were most likely also resistant. In contrast, there are 4 glycan attachments on the HA head of all the more recent strains, including at least one oligomannose attachment [30]. Note that all H1N1 strains since 1977 have the glycan at 104 that is lost in the Braz79/BS strain. Strains isolated in 1978–1980 were highly sensitive to SP-D in our analysis, whereas unexpectedly more recent strains (we tested strains from 1996–2001) were less sensitive. By correlating glycan attachments with sensitivity to SP-D, it appears that glycans at 144 and 172 are important for inhibition by SP-D. These sites were present in the most sensitive strains and are exchanged for sites at 71 and 142 in the more recent, less sensitive strains. Figure 3B shows that the 144 and 172 sites are located closer to the sialic acid binding site than 71 and 142.

We also tested the ability of SP-D to inhibit human H5N1 isolates. Both strains of H5N1 were resistant to SP-D. These strains have 1 or 2 glycans on the HA head but as shown in Figure 3C and Table 2, these are not proximal to the sialic acid binding site.

## Discussion

Most of our prior work on functional interactions of recombinant SP-D with IAV involved the Thr11, Thr160 polymorphic form. We now demonstrate that the Met11, Ala160 form also has strong viral neutralizing activity. In addition, both forms of SP-D cause NA inhibition and virus aggregation and increase neutrophil uptake of the virus. These findings are reasonable given that the trimeric lectin domains of the variants have identical structure.

The recombinant SP-D strongly inhibited recent H3N2 and H1N1 strains of IAV and a recent influenza B strain as well (data not shown). The loss of an oligomannose attachment at 165 in H3N2 caused marked but not complete loss in sensitivity to SP-D as previously reported using other forms of SP-D. Of interest, all wild type strains of H3N2 have this attachment and early studies using the Memphis72 H3N2 strain showed that the sites at 165 and 285 are oligomannose and that at 81 was complex [32]. Hence, despite having the oligomannose site at 165, early strains of H3N2 were at least partially resistant to SP-D. It should be noted that we did obtain slightly different results using avian (Table 2) vs. human (Table 1) erythrocytes in the HA inhibition assay. Using human erythrocytes there was some inhibition of the early H3 strains (Mem71_H_-Bel_N_) but no inhibition was found for comparable strains using avian cells. In any case the overall trends were similar with increased activity against the more recent H3N2 strains.

Abe et al. tested the effects of adding HA head glycans in the naturally occurring sites on the H3 HA on receptor binding and fusion activity and reactivity with antisera [33]. Although the additional glycans reduced receptor binding affinity, they did not alter fusion activity and they reduced the ability of antisera raised against early H3N2 strains to bind to the HA. Similarly, Skehel et al. showed that addition of the glycan at 63 caused loss of reactivity with an inhibitory monoclonal antibody [34]. Hence, one plausible explanation for the accumulation of glycan attachments on the H3 HA is their ability to shield the HA from antibody-mediated neutralization. This may explain the apparently paradoxical trend to increased sensitivity to SP-D. Vigerust et al. have used reverse genetics to test the effect of adding glycans to a 1968 H3N2 strain. Addition of the 63, 126, and 246 sites resulted in a virus more readily inhibited by SP-D and less pathogenic for mice. Further addition of sites at 133 and 144 did not cause a greater increase in sensitivity to SP-D. The overall trend of increased sensitivity with increased glycosylation sites is consistent with our results and with those of Reading et al. [19]. We found the greatest increase in sensitivity to SP-D in strains having glycans at 122, 133 and 144 and show that these sites (especially 133 and 144) are in close proximity to the sialic acid binding site. Putting our results together with those of Vigerust et al, inhibition by SP-D appears to involve having several additional glycan attachments on the HA head apart from the important site at 165.

It is unknown at present the type of glycans present at sites other than 285, 165 and 81. If some are oligomannose this would provide an obvious explanation for their importance in binding to SP-D. If not, another hypothesis is that initial tethering of SP-D occurs by initial binding to the site at 165. Once bound, SP-D can then interact with less favorable sites (e.g, to complex glycans terminated with galactose or hybrid glycans). Another possibility is that the presence of additional glycans reduces binding affinity of the HA to sialic acid receptors rendering it more easy for SP-D to interfere with attachment of the virus to cells. Further studies will be needed to clarify these possibilities.

**Figure 4:**
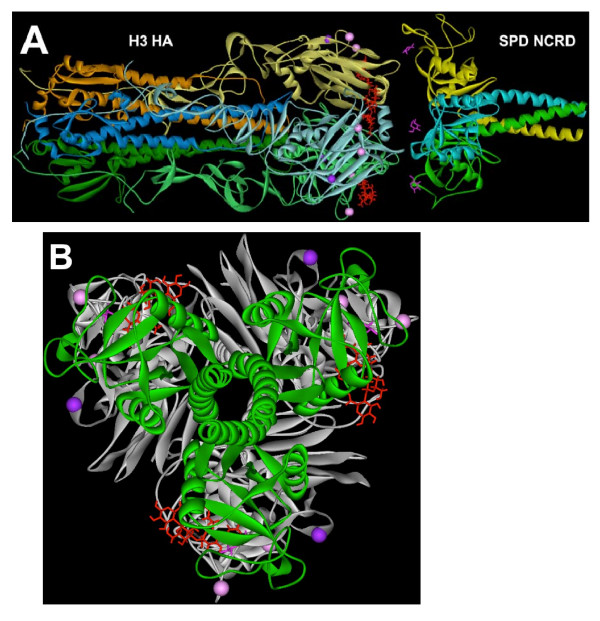
**Alignment of the H3 hemagglutinin (HA) trimer with the neck and carbohydrate recognition domains (NCRD) of an SP-D trimer**. (A) The structure of X31 H3N2 complexed with (a2-3) sialyllactose (red sticks) [38] was aligned with that of the N-terminal carbohydrate recognition domain (NCRD) of SP-D complexed with maltose [40] using Accelrys DS Visualizer 1.7. The distance between the C1 atom of one glucose unit of maltose (dark magenta sticks) and the corresponding Ca atom of position 133 or 144 (light magenta balls) was within 18 to 20 Angstroms for all three monomeric interactions. This distance can be covered by an asparagine side chain plus three or more glycan units, suggesting that a glycan originating from either position 133 or 144 of HA can fit into the glycan binding pocket of SP-D and simultaneous interaction by all three monomers are possible. The Ca atoms of position 122 (purple balls) are too far (~30 angstroms) from the corresponding the C1 atoms of glucose units bound to SP-D, and unlike positions 133 and 144, the path of 122 to that glucose is blocked by a fold in the hemagglutinin structure. Each monomer of HA and SP-D is shown as ribbons with different colors matching each monomeric interaction. (B) The side view of the aligned HA and SP-D trimers in (A) was turned approximately 90 degrees to generate an axial view from the SP-D stem in which the trimeric hemagglutinin is shown as white ribbons while the trimeric SP-D is depicted as green ribbons. Binding by SP-D to the glycans originating at the Ca atoms at position 133 or 144 (light magenta balls) of hemagglutinin would completely block the ability of HA to bind to cellular sialic acid targets (red sticks). Alignment of the H1 hemagglutinin based on its positions 144 and 172 onto the NCRD of SP-D yielded similar conclusions.

Using crystal structures coordinates of the H3 and SP-D trimers, Figure 4A demonstrates that when these trimers are aligned along their three-fold axes, the carbohydrate recognition domains of the SP-D trimer are positioned to interact with one or both of the glycans at positions 133 and 144 on the HA head, consistent with the deductions from the hemagglutination data that these two glycan are involved in the sensitivity of the H3N2 strains to inhibition by SP-D. This alignment also demonstrates that the glycan at position 122 is less likely to be important for interacting with SP-D. Figure 4B shows that the alignment of the carbohydrate recognition domain of SP-D with the glycans at 133 and 144 results in the masking of the sialic acid binding site of HA (red), providing a molecular level explanation for the sensitivity of the H3N2 strain to hemagglutination inhibition by SP-D. Analogous structures and conclusions were obtained for H1N1 and residues 144 and 172 (data not shown).

Although the alignment shown in Figure 4 represents a possible interaction between SP-D and HA trimers, it is important to note that other interactions are also possible. For example, it is possible that a single SP-D trimer could act as a "bridge" between adjacent HA trimers on the viral envelope (or even possibly between HA and NA). It is also possible that other glycans on HA could be accessed by a single arm of the SP-D trimer. However, given the nearly perfect alignment of carbohydrate recognition domains of the SP-D trimer with glycans 133 and 144 of the HA trimer Figure 4A), suggests that interactions of this type are energetically most favorable.

We could not test H2N2 strains, but it is of interest that these strains had only 2 glycans on the HA head (neither of which was oligomannose) [35] and may, therefore, have been resistant to SP-D. The H2 HA was limited in its ability to add additional glycans to the head since added glycans not only inhibited receptor binding but also fusion and infectivity [36]. It is speculated that this limitation may have restricted the ability of H2N2 strains to survive longer in the human population since they could not protect themselves against antibody attack. The H3N2 lineage seems to have been able to achieve a better balance in which strains are protected from antibody-mediated neutralization (and possibly attenuated in their severity somewhat by virtue of increased sensitivity to SP-D) allowing persistence over a longer period in the human population.

The H1 lineage is also somewhat distinctive from the H3 and H2 lineages. Originally the 1918 strains (like most duck and swine strain H1N1 strains) [37] lacked glycans on the HA head. The re-emergence of H1N1 in 1977 was associated with the addition of 4 new glycan attachments on the HA head. Since then the human H1N1 lineage has continued to have 4 such glycans but their locations have shifted (i.e., sites at 144 and 172 were lost and sites at 71 and 142 added). The site at 104, like that at 165 in the H3N2 lineage, is apparently important for tethering of SP-D but other glycans on the HA head are involved in interactions with SP-D. Based on our results it appears that attachments at 144 and 172 are more effective in mediating effects of SP-D than 71 and 142 and this fits with the relative proximity of these sites to the sialic acid binding site on the HA. Reverse genetic analysis of the role of specific glycans on the H1 HA, combined with studies of sensitivity to collectins and antibodies, would be of great interest.

It is of potentially great importance that the H5N1 strains were not susceptible to SP-D (and that the 1918 H1N1 strain is predicted to not be sensitive) since this could be one factor accounting for the clinical severity of infection with these strains.

## Conclusion

Overall our results show that the Met11 form of SP-D has strong antiviral and opsonizing activities. Using this preparation we show that there is a correlation between glycan attachments on the head of the HA and the susceptibility of IAV to inhibition by SP-D that implicates specific sites on the HA as important in binding to SP-D. Of note, pandemic strains (e.g., strains of H3N2 and H1N1 isolated shortly after introduction into the human population) and human H5N1 strains are largely resistant to SP-D. The H3 HA has evolved to have persistently increased HA glycosylation and sensitivity to SP-D, whereas the H1 HA showed greater sensitivity in the 1970's and 1980's and has become less sensitive despite maintenance of 4 glycan sites on the HA. Further studies of H5N1 or H1N1 subtype viruses using reverse genetics to modify HA glycan sites should be undertaken to more precisely determine the role of specific glycans in susceptibility to SP-D.

## Competing interests

CP, SB, RJM, and RKS hold shares and/or stock options of Genzyme Corporation. KLH, MRW, TT, and ECC have no competing interests.

## Authors' contributions

KLH participated in writing the manuscript, designing experiments and interpreting results. RW carried out the hemagglutination assays. MRW carried out some of the hemagglutination inhibition assays (Table 1) and assays in figure 2. TT performed some of assays shown in figure 2. CP carried out the structural alignments of SP-D and influenza HA. SB characterized the rhSP-D by AUC and other methods. RJM helped clone and produce the rhSP-D and participated in experimental design. ECC assisted in the experimental plan, interpretation of results and editing of the manuscript. RKS conceived the study, designed and coordinated experiments, interpreted results and helped write the manuscript. All authors read and approved the final manuscript.
